# Effects of hydraulic retention time on adsorption behaviours of EPS in an A/O-MBR: biofouling study with QCM-D

**DOI:** 10.1038/s41598-017-03190-1

**Published:** 2017-06-06

**Authors:** Xudong Wang, Botao Cheng, Cunrui Ji, Miao Zhou, Lei Wang

**Affiliations:** Key Laboratory of Membrane Separation of Shaanxi Province, School of Environmental & Municipal Engineering, Xi’an University of Architecture and Technology, Yan Ta Road. No.13, Xi’an, 710055 China

## Abstract

Extra-cellular polymeric substances (EPS) are a major cause of membrane fouling in membrane bioreactors (MBRs). In this study, an anoxic–oxic membrane bioreactor (A/O-MBR) was run continuously for 98 days. The runs were divided into three stages according to hydraulic retention time (HRT) (11.8, 12.5 and 14.3 h, respectively). EPS were extracted from the reactor under the different HRTs. A quartz crystal microbalance with dissipation monitoring (QCM-D) and Fourier transform infrared (FT–IR) were used to study the adherence layer structures and the adsorption behaviours of EPS on the membrane surface. The results indicated that the removal rate of TN was more susceptible to HRT than NH_3_-N. The observations in the QCM-D suggested that at the lowest HRT (11.8 h), the structure of the adsorption layer is loose and soft and the fluidity was better than for HRTs of 12.5 or 14.3 h. It is likely one of the major reasons for the rapidly blocking of the membrane pores. Furthermore, the higher EPS adherence as analyzed in the QCM-D and EPS concentration could induce a higher osmotic pressure effect, leading to a rapid membrane-fouling rate.

## Introduction

Membrane fouling is a major obstacle to using membrane bioreactors (MBRs) because fouling increases the transmembrane pressure (TMP) and decreases the permeate flux^1^. The economical operation of an MBR requires in-depth analysis of membrane fouling. In MBR processes, the membrane properties, operational conditions such as hydraulic retention time (HRT), and biomass characteristics such as extra-cellular polymeric substances (EPS) will affect the membrane fouling^2^.

EPS are the construction materials for microbial aggregates such as biofilms and activated sludge flocs. EPS can be divided into bound EPS and soluble EPS (also called soluble microbial products [SMP]), both of which include bacterially produced polymers, lysis products, and hydrolysis products. Bound EPS are dissolved or hydrolyzed by bacterial hydrolysis, while soluble EPS are biodegradable and a product of the dissolution of bound EPS. They consist of several classes of polysaccharides, proteins, humic substances, nucleic acids, lipids, and other polymeric compounds, and have been found at or outside the cell surface and in the intercellular space of microbial aggregates^3, 4^. EPS can strongly affect the surface charge, hydrophobicity or hydrophilicity, and adhesion ability of sludge flocs, and the dynamic viscosity of the mixed liquor^5^. EPS play a major role in the cohesion of sludge flocs in the MBR and its viscoelasticity can strongly affect the resistance of the flocs and the biofouling layer to shear^6, 7^. Consequently, EPS are regarded as a significant factor affecting biofouling in membrane bioreactor^8^. It has been shown that low bound EPS content inhibit the self-accelerating phenomena leading to a TMP jump, thus permitting a longer sustainable filtration operation^9^. Soluble EPS could be readily deposited and adsorbed on and/or into the membrane, form a gel layer, then cause membrane pore blocking, and penetrate into the pores and spaces between particles in the cake layer. The gel layer had unusually high specific filtration resistance being almost 100 times higher than the cake layer^10^.

One of the most effective MBR operating parameters with an impact on fouling propensity is HRT, which affects various sludge properties such as floc size, bound and soluble EPS content, and settling characteristics^11^. The effects of different HRTs on membrane fouling and biomass characteristics in submerged-membrane bioreactors were investigated by Meng *et al*. for synthetic wastewater treatment, who found that lower HRT (4–5 and 6–8 h) caused excessive growth of filamentous bacteria than higher HRT (10–12 h), which resulted in high EPS concentration, high mixed liquor suspended solids (MLSS) concentration, and high sludge viscosity^12^. Huang *et al*. operated three lab-scale submerged anaerobic MBRs with solids retention times (SRTs) of 30, 60 and infinite days were setup for treating synthetic low-strength wastewater at HRTs of 12, 10 and 8 h. The results suggested that a decrease in HRT (10 or 8 h) enhanced the growth of biomass and accumulation of SMP, which accelerated the membrane-fouling rate^13^. Deng *et al*. examined the membrane-fouling potential in sponge-submerged MBRs operated at different HRTs (6.67, 5.33 and 4.00 h) for synthetic wastewater treatment. They found that at shorter HRTs, more obvious membrane fouling was caused by exacerbated cake layer formation and aggravated pore blocking. Increased HRT could alleviate cake layer formation and prevent pore blocking, thereby minimizing membrane fouling^6^. Another study conducted by Shariati *et al*. examined the effects of HRTs (8, 16 and 24 h) on the performance of membrane sequencing batch reactor (MSBR) for the treatment of synthetic petroleum refinery wastewater. The rate of membrane fouling was found to increase with decreasing HRT, carbohydrate SMP, and mixed liquor apparent viscosity also showed a pronounced increase with decreasing HRT^14^. However, most of these studies used synthetic wastewater as the substrate. Given some obvious difference such as viscosity, trace element and inevitable bacteria-inhibitor, it is more practical to use actual sewage than synthetic wastewater^15^. Babatsouli *et al*. operated an MBR pilot plant with a short SRT of 20 d for industrial Park sewage treatment. A sudden increase in TMP was observed after HRT reduced from 24 to 19 h, which led to a higher flux resulting in a higher rate of fouling^16^.

The accumulation of EPS on the membrane surface is a complex process that is affected by matrix composition, operating pressure, organic loading rate, MLSS concentration, SMP composition, and membrane properties^17, 18^. The adhesion forces of membrane–humic acid (membrane–HA) and HA–HA at pH 3, 7, 11 were measured by atomic force microscopy (AFM), respectively. The results of AFM force measurements illustrated that the adhesion force in acidic environment was much stronger than that in alkaline or neutral environment, and the adhesion force of PVDF/polyvinylalcohol membrane–HA (PA–HA) was weaker than that of PVDF/polyvinylpyrrolidone membrane–HA (PP–HA)^19^. Attenuated total reflection–fourier transform infrared spectroscopy (ATR–FTIR) was used to show the functional groups of fouling species by Zhou *et al*.^20^. They prove that the amide I (C = O) and amide II (C–N + N–H) bands were existed in proteins. Ivnitsky *et al*. based on polymerase chain reaction–denaturing gradient gel electrophoresis (PCR–DGGE) method to analyze the bacterial community composition and structure of biofilms developing on membranes surface. Deposition of polysaccharides and initial bacterial colonization were observed within 8 h, whereas developed biofilms markedly affecting membrane permeability were evident from days 2–3 onwards. *Pseudomonas/Burkholderia*, *Ralstonia*, *Bacteroidetes* and *Sphingomonas* were the dominant bacterial populations groups found in most biofilms^21^. But, these studies lack a direct method to characterize the adsorption process of contaminants on the membrane surface. Therefore, the direct membrane autopsy and analysis of the accumulated EPS should help to relate EPS properties and membrane fouling. The Quartz Crystal Microbalance with Dissipation (QCM-D) is an acoustic surface-sensitive technique (ng/cm^2^ sensitivity) that provides simultaneous, real-time information on mass, structure of molecular layers, and label-free measurements of molecular adsorption and/or interactions taking place on various surfaces^22, 23^. The QCM-D technique has been employed to study the viscoelasticity and adherence of EPS^24^, collagen adsorption^25^, and deposition kinetics of bacteria^26^. In essence, QCM measures the amount of adhering mass by means of shifts in the resonance frequency (Δ*f*) of an oscillating quartz crystal sensor. In addition, the amplitude of oscillation is influenced by dissipative energy losses caused by the viscoelastic properties of the adsorbed film. These energy losses can be quantified from the frequency bandwidth or the oscillation decay time (dissipation, Δ*D*)^27^.

Bearing the information above, the further research is needed to evaluate the membrane fouling process for actual sewage treatment. In this study, we mainly focused on the effects of adherence layer structure and the adsorption behaviours of the EPS on membrane fouling at different HRTs for actual sewage wastewater treatment. QCM-D monitoring and Fourier transform infrared (FT–IR) were employed to characterize the pollution process of EPS on the membrane surface. EPS were extracted from an A/O-MBR reactor operated at different HRTs. The TMP changes were used to characterize the flux variation.

## Results and Discussion

### Effect of HRT on the operation performance of the A/O-MBR

There were three runs during the operation process according to the HRT: (i) the run 1 was from day 1 to day 38 with HRT at 11.8 h, which include a start-up stage (day 1 ~ 21), where the MBR were unstable; (ii) the run 2 was from day 39 to day 68 with HRT at 12.5 h; and (iii) the run 3 was from day 69 to day 98 with HRT at 14.3 h.

**Figure 1:**
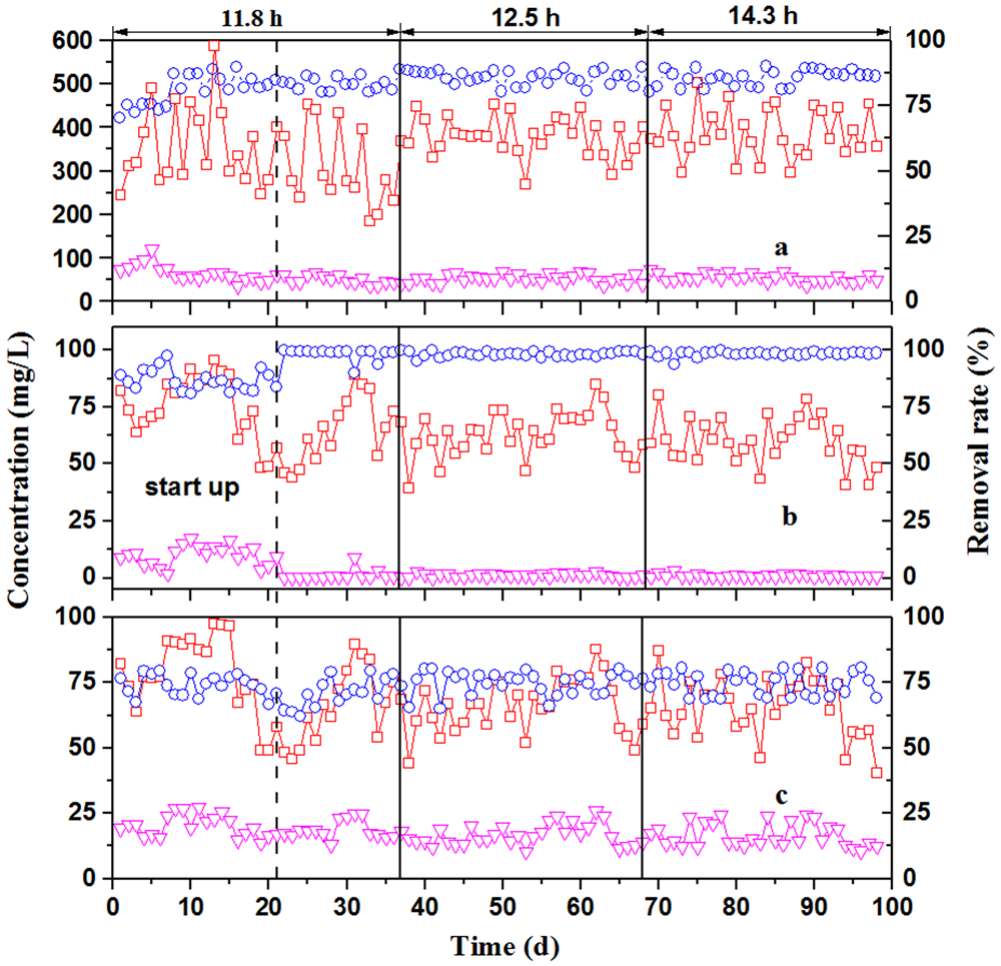
Treatment performance of the A/O-MBR: (**a**) COD; (**b**) NH_3_-N; and (**c**) TN. **□**: Influent concentration, ∇: effluent concent ration, and **⚪**: removal rate.

The system performance in terms of COD, NH_3_-N, and TN at different HRTs and influent concentrations are shown in Fig. 1. The removal rates are also summarized in Table 1. Regardless of the variation in raw wastewater (COD = 134.6–587.5 mg/L) during the whole process, the average effluent COD values were 49.7 ± 9.2, 53.4 ± 9.0 and 54.9 ± 8.8 mg/L for HRTs of 11.8, 12.5 and 14.3 h, respectively (Fig. 1a). The results suggest that increased HRT has little effect on removal of COD. During the test, NH_3_-N influent values varied in the range 39.3–95.68 mg/L (Fig. 1b). Because of the long generation time of nitrifying bacteria, the removal rate of NH_3_-N at 86.7 ± 4.6% fluctuated during the start-up stage. After the reactor operated stably, it achieved more than 98% of NH_3_-N removal and the effluent values stabilized at 1.1 ± 0.2, 1.2 ± 0.7 and 1.0 ± 0.7 mg/L for HRTs of 11.8, 12.5 and 14.3 h, respectively. The mean influent concentration of TN was 67.0 mg/L and the change of operation conditions affected the removal rates of TN, which were 71.1 ± 5.5%, 74.9 ± 4.4% and 74.5 ± 4.6%, respectively (Fig. 1c).

**Table 1:** System performance at different HRTs.

Time (day)	HRT (h)	Influent (mg/L)			Effluent (mg/L)			Removal rate (%)		
		COD	NH_3_-N	TN	COD	NH_3_-N	TN	COD	NH_3_-N	TN
1–21	Start up	357.3 ± 90.9	75.1 ± 13.9	78.6 ± 14.8	65.4 ± 18.8	10.1 ± 4.3	20.2 ± 4.2	81.0 ± 5.9	86.7 ± 4.6	74.0 ± 4.0
22–38	11.2	310.3 ± 87.9	65.0 ± 14.2	66.4 ± 14.0	49.7 ± 9.2	1.1 ± 0.2	18.8 ± 3.5	83.4 ± 2.6	98.4 ± 2.7	71.1 ± 5.5
39–68	12.5	379.1 ± 45.4	62.7 ± 10.0	66.1 ± 10.3	53.4 ± 9.0	1.2 ± 0.7	16.6 ± 4.0	85.8 ± 2.7	98.1 ± 1.1	74.9 ± 4.4
69–98	14.3	386.7 ± 55.6	60.0 ± 10.2	65.3 ± 11.2	54.9 ± 8.8	1.0 ± 0.7	16.7 ± 4.5	85.5 ± 3.0	98.3 ± 1.1	74.5 ± 4.6

Note: the concentrations in influent, effluent and the removal rate were calculated as the average values during the whole experiments.

### Effect of HRT on the filtration performance of the A/O-MBR

Related research shows that the TMP changes in the process of membrane fouling can be divided into three stages^28, 29^. Stage I occurs over the first few hours and involves an abrupt TMP rise because of bacterial adhesion to the membrane surface, eventually leading to membrane pore blockage and closure. Stage II is a long-term slow rise in TMP as EPS, colloids, and other products of bioactivity are adsorbed slowly onto the membrane surface; the foulants are produced by the sludge mixture and the biofilm. Stage III is a sudden rise in TMP, which rapidly leads to inoperability of the membrane. This sudden jump is possibly not only because of the local flux effect, but also because of sudden changes in the biofilm or cake layer structure.

**Figure 2:**
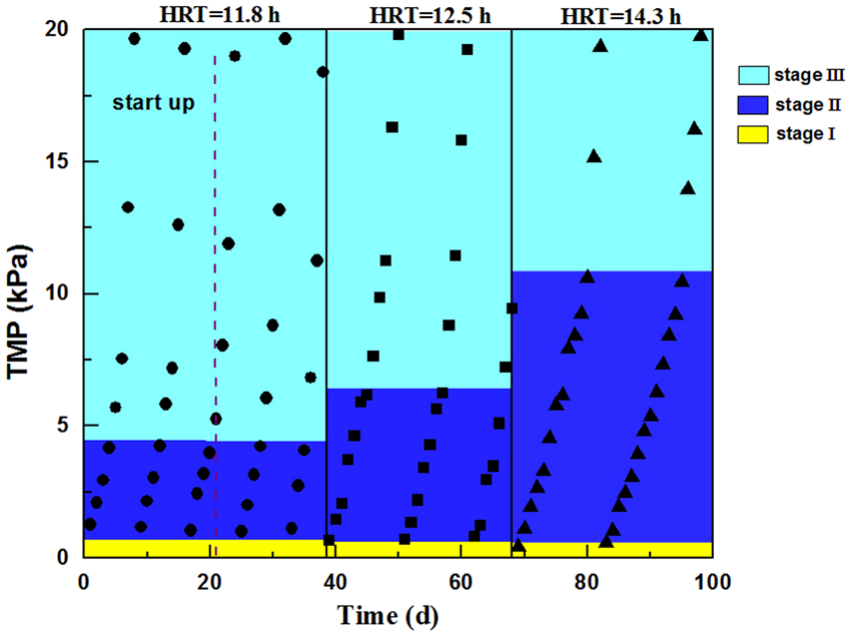
The variation of TMP against operation time under different conditions. Yellow area represents the stage I of TMP changes, Blue area represents the stage II of TMP changes, Cyan area represents the stage III of TMP changes.

TMP was monitored every day (Fig. 2). After the reactor stably, at the HRT of 11.8 h (day 22 ~ 38), the TMP increased most rapidly, and the membrane-fouling rate was the fastest. The stage I of TMP changes occurs over the first few hours in the first day at each cleaning cycle and involves an abrupt TMP changes. The stage II of TMP changes was characterized by a slow TMP increase from approximately 1.0 kpa to 4.2 kpa during every cleaning cycle. A jump in TMP was observed at about day 5 of each cleaning cycle, when the average TMP increase changed from 1.34 kpa/d to 5.63 kpa/d. At the HRT of 14.3 h, the integral TMP increase was relatively slower than those at 11.8 and 12.5 h. The stage II of TMP increased from approximately 1.1 kpa to 7.2 kpa during every cleaning cycle. The jump in TMP appeared at day 13 of each cleaning cycle, when the average increase in TMP changed from 0.87 kPa/d to 3.73 kPa/d. The EPS in MBR has a great influence on membrane-fouling rate^30^. Therefore, further analysis of EPS at different HRTs with QCM-D was conducted using the A/O-MBR system.

### Effect of HRT on EPS adherence and adsorption behaviours

**Figure 3:**
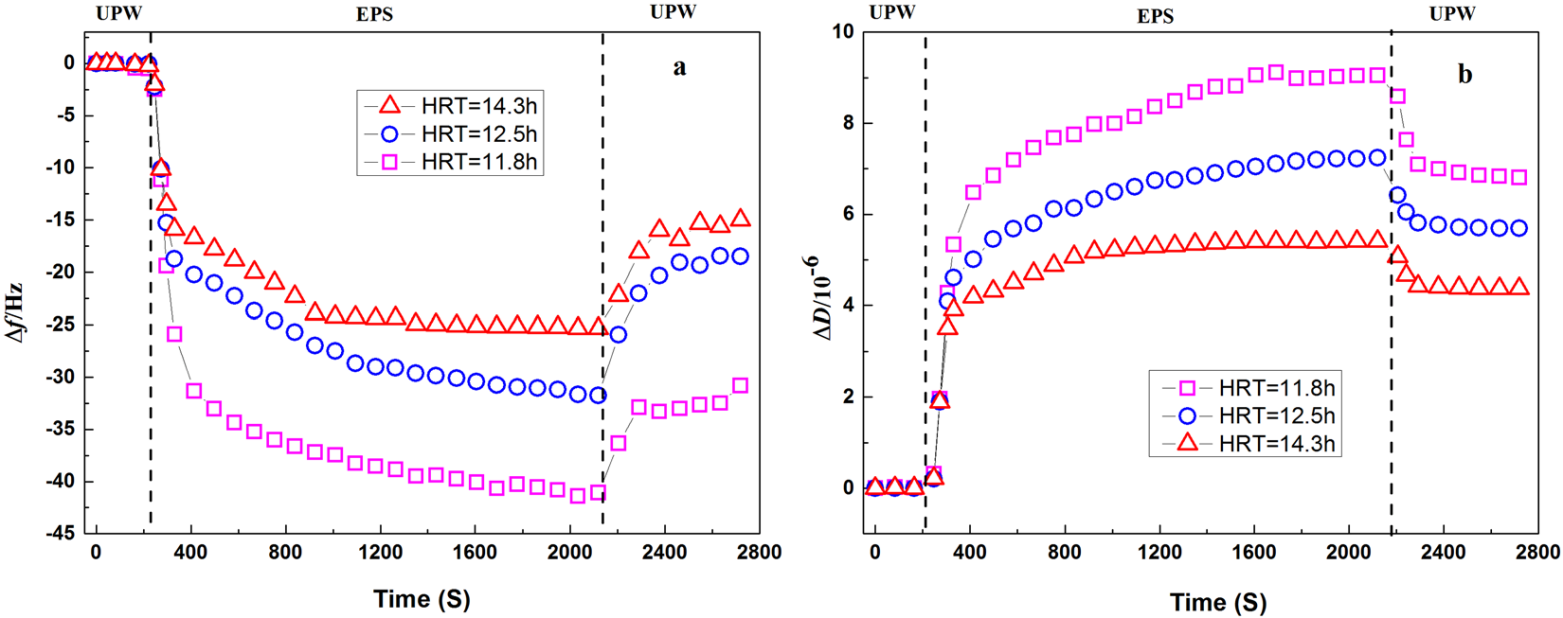
EPS adherence properties, extracted from the reactor, after runs operated at different HRTs. Frequency shifts (**a**) and dissipation factors (**b**) during EPS adsorption to PVDF-coated QCM-D sensors.

EPS adherence and adsorption behaviours were analysed during adsorption to PVDF-coated crystals in a QCM-D at different HRTs. Figure 3(a,b) shows the decrease in frequency and increase in dissipation energy of the PVDF crystal caused by adsorption of EPS originating from the A/O-MBR operated at different HRTs. The highest EPS adsorption rate showed as a decrease in the PVDF-coated crystal frequency was observed for the EPS extracted from the reactor at HRT of 11.8 h while the lowest EPS adsorption rate was observed for the EPS originated from MBR operation at HRT of 14.3 h. It is clear that the adsorption of EPS on the sensor crystal surface goes through two stages when the EPS first enters the flow cell. In the initial stage (approximately from 200 to 1800 seconds), EPS is attached to the membrane surface quickly and forms a dense pollution layer, with a sharp increase in |Δ*f*|, indicating that the first stage of membrane pollution is more rapid. Previous studies revealed that this process is mainly affected by the interaction forces of EPS molecules and membrane materials, such as Lifshitz-van der Waals (LW), acid-base (AB), and electrostatic double layer (EL) interaction forces. Under the interaction of three types forces will produce an energy barrier, only EPS with energy higher than the energy barrier can eventually adhere to membrane surface^31, 32^. A similar trend was observed under different HRTs while investigating the deposition behaviour of EPS on a sensor crystal surface; however, at the HRT of 11.8 h, the Δ*f* changes are the largest, indicating that at smaller HRTs, the EPS adherence was higher, membrane fouling was more serious. When the adsorption of EPS on the membrane surface enters the second stage, near steady-state Δ*f* values for each EPS solutions were reached within approximately 1800–2200 seconds.

**Figure 4:**
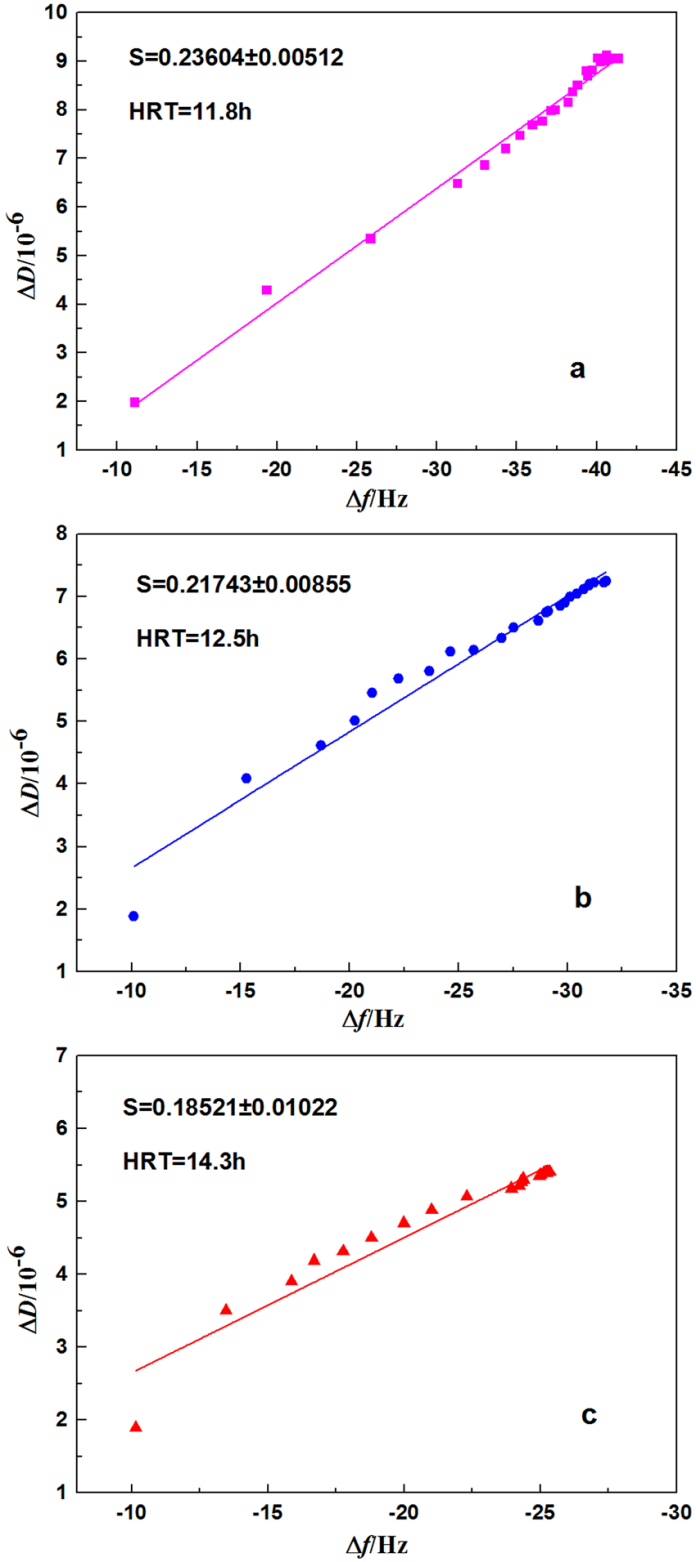
Comparison of the fluidity of different EPS extracted from the reactor after runs operated at different HRTs: (**a**) 11.8 h; (**b**) 12.5 h; and (**c**) 14.3 h. Dissipation factors versus frequency shifts during adsorption are shown. *S* shows the slope of the linear approximation.

Δ*D* mainly reflects the changes in the energy dissipation of the sensor crystal. To obtain a deeper understanding of the contaminant adsorption layer, the |Δ*D*/Δ*f*| ratio is usually used to characterize the structural information of the EPS adsorption layer on the membrane surface. A high |Δ*D*/Δ*f*| ratio corresponds to a relatively loose and soft structure; a low ratio corresponds to a stiffer, more compact structure, in which the adsorbed mass induces relatively low energy dissipation^33^. The slope of |Δ*D*/Δ*f*| can be used to characterize the fluidity of the adsorption layer; larger slope values indicate that the film attached to the surface is more fluid and viscoelastic^34, 35^. The slopes of Δ*D*/Δ*f* for each HRT are shown in Fig. 4(a,b,c). The trends observed for the changes in slope show an interesting behaviour; at the lowest HRT of 11.8 h, the extracted EPS layers are more fluid than the EPS layers extracted from the reactor exposed to higher HRTs of 12.5 and 14.3 h. This result suggests that the pollution at HRT of 11.8 h is more rapid with a higher EPS fluidity (Fig. 4a). Sweity *et al*. found that EPS fluidity and swelling induced at high pH make major contributions to pore clogging^36^. And in addition to a higher EPS adherence, the fluidity of the EPS fouling layer, covered on the PVDF-coated sensor crystal surface, is likely playing an important role in its accessibility to the membrane pores that eventually are being accumulated more rapidly by the EPS^24^. In this study, the structure of the EPS fouling layer was loose and soft at the lowest HRT (11.8 h) showed the strongest fluidity as analyzed in the QCM-D, it is likely one of the major reasons for the rapidly blocking of the membrane pores.

### Relation between EPS composition, adherence, and membrane-fouling rate

**Figure 5:**
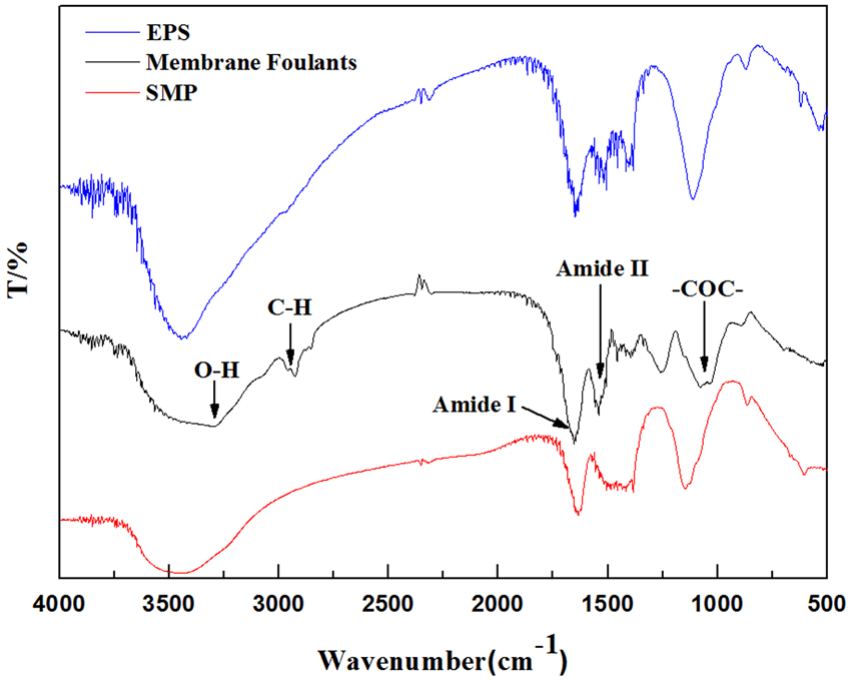
FT–IR spectra of the membrane foulants, SMP and EPS.

FT–IR is a powerful method for characterizing the functional groups of organic matter^37, 38^. The FT–IR spectra of membrane foulants SMP and EPS are shown in Fig. 5. A broad region of the adsorption spectrum of membrane foulants was found at 3300 cm^−1^, which reflected stretching vibrations of O–H bonds, and a sharp peak was also present at 2900 cm^−1^, which reflected stretching vibration of the aromatic C–H bond. There are two peaks at 1650 cm^−1^ and 1540 cm^−1^, called Amides I and II, in the spectrum that are unique to protein secondary structures^39, 40^. The Amide I peak is caused by the stretching vibration of the C = O bond in the peptide groups, while the Amide II peak is a combination of N–H bending and C–N stretching^26^. In addition, there is another obvious peak at 1100 cm^−1^, which represents -COC- wagging, indicating the presence of polysaccharides and polysaccharide-like substances^41^. The spectra indicate that proteins and polysaccharides are the major components of membrane foulants. From the EPS and SMP spectra (Fig. 5), contrast analysis shows that the EPS and SMP in the membrane pool all had organic functional groups similar to those of the membrane foulants. However, the SMP mainly contains polysaccharides and humic acids and the protein peptide bond band is weaker than that of EPS. The absorption spectrum of EPS was similar to that of the membrane foulants, and the similar organic matter content in EPS is the highest. This result is consistent with that of Jarusutthirak *et al*.^42^, who studied the effect of SMP and EPS on the critical fluxes in MBRs.

**Figure 6:**
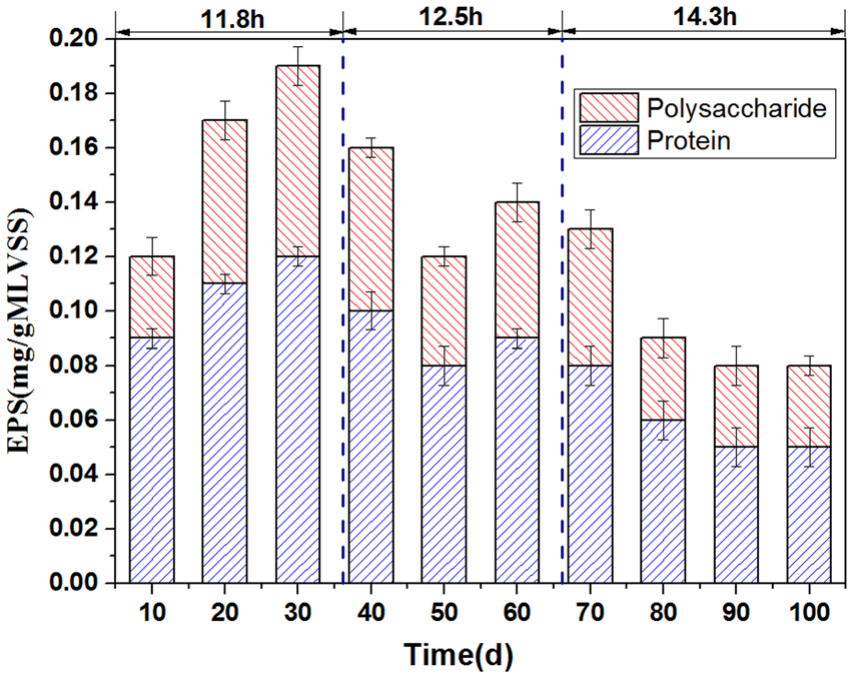
Change of EPS against operation time. Note: the EPS concentrations were shown in forms of ‘average value ± standard deviation’.

Polysaccharide (PS) and protein (PN) are known to be two primary components of EPS. The changes in EPS content are shown in Fig. 6. It is clear that the EPS concentrations extracted from the reactor are higher for HRT of 11.8 h than for 12.5 and 14.3 h. In general, cake layer formation is the main cause of membrane fouling in submerged MBR (SMBR)^43, 44^. It was reported that the cake layer formed on membrane surface was rich in EPS and negatively charged, which will lead to an osmotic pressure that existed during cake layer filtration process, the osmotic pressure effect is the major contributor of total cake resistance^45, 46^. Therefore, the higher EPS adherence (Fig. 3a) as analyzed in the QCM-D and higher EPS concentrations (Fig. 6) at HRT of 11.8 h could induce a higher osmotic pressure, leading to a rapid membrane-fouling rate (as show in Fig. 2).

## Conclusions

In this study, the QCM-D technique was the main tool used to analyse the adherence layer structure and fluidity of the EPS in an A/O-MBR. The following conclusions were obtained. (i) The effect of HRT on the effluent COD was small. When the system was running stably, the NH_3_-N removal rate exceeded 98%. The effect of HRT on the removal rate of TN was greater than that on NH_3_-N. (ii) FT–IR analysis showed that PS and PN were the main components of membrane foulants. From the EPS/SMP analysis, we found that the absorption peak of EPS was similar to that of the membrane foulants, which indicates that EPS is the main pollutant in the reactor. (iii) The EPS adherence layer structure and the adsorption behaviours on the membrane surface can be described visually using the QCM-D technique. EPS is attached to the membrane surface quickly to form a dense pollution layer, followed by further EPS deposition on the membrane, near steady-state Δ*f* values for each EPS solutions were reached. At the lowest HRT (11.8 h), the structure of the adsorption layer is loose and soft and the fluidity was better than for HRTs of 12.5 or 14.3 h, it is likely one of the major reasons for the rapidly blocking of the membrane pores. Furthermore, the higher EPS adherence as analyzed in the QCM-D and EPS concentration could induce a higher osmotic pressure effect, leading to a rapid membrane-fouling rate.

## Materials and Methods

### A/O-MBR system and operating conditions

**Figure 7:**
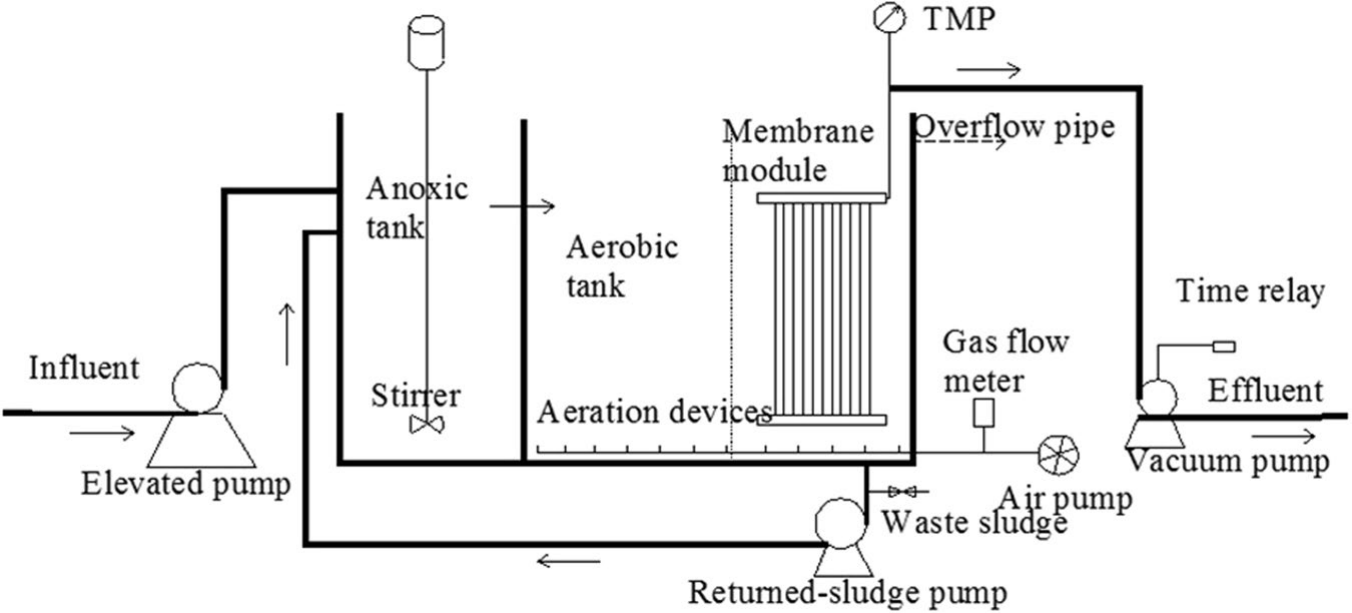
Schematic design of the A/O-MBR.

The bench-scale A/O-MBR system was consisted of an activated sludge bioreactor and an immersed UF membrane module (Fig. 7). The activated sludge used was from the fifth municipal treatment plant in Xi’an and the experimental wastewater was real domestic wastewater with the quality shown in Supplementary Table S1. The SRT of this A/O-MBR was 30 days for all the experiments. The membrane flux was set at approximately 10 L/(m^2^ h). The membrane modules were cleaned using an *ex situ* cleaning method when the TMP reached 20 kPa, using the sequence of 0.05% sodium hypochlorite for 1 h and 1% citric acid for 1 h as a cleaning cycle.

The membrane used in this experiment was a polyvinylidene fluoride (PVDF) hollow fibre membrane with a mean pore size of 0.1 μm and a total effective filtering surface area of 0.1 m^2^. This experimental membrane was made in our laboratory from high-strength SiO_2_ modified with PVDF/PET. The basic parameters of the composite membrane are shown in Supplementary Table S2. The bioreactor had an effective volume of 15 L. Raw water was pumped to the anoxic tank using a peristaltic pump, and then flowed via a perforated baffle to the aerobic pond and after settling, the slurry mixing liquid flowed back to the anoxic tank via the return sludge pipe. The gap between the membrane and the wall was set at 7 mm to obtain efficient scouring of the membrane by the airflow. The microporous aeration head was fixed 5 cm from the bottom and the membrane aeration rate was 7 L/min. The water peristaltic pump was controlled by a time relay.

### EPS extraction and analysis

The extraction of EPS was based on cation exchange using a cation-exchange resin (CER) (Dowex, Na^+^-form, 20–50 mesh, Sigma-Aldrich 91973). This extraction procedure was based on the method by Frølund *et al*.^47^. Activated sludge thickened to an MLSS concentration of 8 g/L was used as the sludge sample. The sludge sample was centrifuged at 2000 *g* for 5 min at 4 °C and the supernatant liquid removed. The remaining sludge in the centrifuge tube was resuspended to the original volume using a buffer solution and then centrifuged. This sludge washing process was repeated three times. The washed sludge was transferred to an extraction beaker with baffles and the CER, which had been washed previously for 1 h in extraction buffer solution, was added (75 g/g VSS). The suspension was stirred at 900 rpm for 4 h at 4 °C. The extracted EPS was harvested by centrifugation at 12,000 *g* for 15 min and filtration through a 0.45 μm acetate–cellulose membrane for polysaccharide (PS) and protein (PN) measurements. PS and PN were analysed using the phenol–sulfuric method^48^ and the Coomassie brilliant blue G-250 method^49^, respectively.

### Water quality measurements

Determinations of COD, NH_3_-N, and TN were made according to standard methods^50^.

### QCM-D analysis

A quartz crystal microbalance with dissipation monitoring (QCM-D, E1, Q-Sense, Sweden) was used to analyse the adherence and adsorption behaviours. The gold-coated sensor crystal (QSX301 Au, Q-sense) was coated with a PVDF membrane using the following method^51^: (i) the gold-coated sensor crystal was soaked in a volume ratio of 5:1:1 in ultrapure water (UPW), 25% aqueous ammonia, and 30% hydrogen peroxide cleaning solution for 15 min at 75–80 °C, then dipped in UPW for 5 min, rinsed thoroughly with UPW and dried with pure N_2_ gas; (ii) a homogeneous PVDF solution was prepared by dissolving PVDF in DMAC; and (iii) the cleaned gold-coated sensor crystal was coated with PVDF solution, and a PVDF membrane was formed on the surface of the sensor crystal. The PVDF-coated sensor crystal was rinsed thoroughly with UPW and then dried under pure nitrogen gas for about 60 s.

Prior to the QCM-D experiments, a new PVDF-coated sensor crystal was mounted on a QCM-D quartz flow cell. A baseline with UPW was acquired, and then the EPS solutions were injected sequentially into the QCM-D system with a flow rate of 0.1 ml/min above the sensor surface for 30 min. The temperature was set at 23 °C. The variations of frequency, Δ*f* (Hz), and dissipation factor, Δ*D*, were measured for four harmonics (*n* = 3, 5, 7, and 9). The relationship between the crystal sensor frequency Δ*f* and the mass adhering to the sensor crystal surface followed the Sauerbrey law^52^:

1 $${\rm{\Delta }}m=-\frac{C}{n{\rm{\Delta }}f},$$

where Δ*m* is the mass adsorbed on the sensor, *n* is the harmonic number, and *C* is the crystal constant (17.7 ng·Hz^−1^·cm^−2^). Dissipation could be used to reflect the energy dissipation of the adsorbed material during deposition, which could provide insight into the structure of the deposited EPS.

## Electronic supplementary material

Supplementary information
