# Supplementary material for: Effects of hydraulic retention time on adsorption behaviours of EPS in an A/O-MBR: biofouling study with QCM-D
Source: Sci Rep. 2017 Jun 6;7:2895. doi: 10.1038/s41598-017-03190-1 (PMC5460173; doi:10.1038/s41598-017-03190-1)
Supplement: Supplementary file 1 — Supplementary information [file 41598_2017_3190_MOESM1_ESM.doc]

**Supplementary Information**

**Effects of hydraulic retention time on adsorption behaviours of EPS in an A/O-MBR: biofouling study with QCM-D**

Xudong Wang, Botao Cheng, Cunrui Ji, Miao Zhou, Lei Wang*

Key Laboratory of Membrane Separation of Shaanxi Province, School of Environmental & Municipal Engineering, Xi’an University of Architecture and Technology, Yan Ta Road. No.13, Xi’an 710055, China

*Corresponding author email: wl0178@126.com & xudongw7904@163.com

Tel: +86 029 8220 2729

Fax: +86 029 8220 2729

**Table S1. Raw wastewater quality**

| Parameter | Numerical | Parameter | Numerical |
| --- | --- | --- | --- |
| COD/ (mg/L) | 134.64~587.52 | Temperature/℃ | 11~23 |
| NH_3_-N/ (mg/L) | 39.3~95.68 | TOC/ (mg/L) | 28.90~47.49 |
| TN/ (mg/L) | 44.02~97.63 | Total Coliform Group  /A·（100ml）^-1^ | 3.8×10^7^ |
| Turbidity/NTU | 155~200 | pH | 6. ~7.6 |

**Table S2. The parameters of composite membrane**

| Parameter | Outer diameter  /mm | Coating thickness  /mm | Membrane pore  /nm | Porosity  /% | Tensile Strength  /MPa | Pure Flux  / L/(m^2^∙h) | |
| --- | --- | --- | --- | --- | --- | --- | --- |
| Numerical | 2.1±0.05 | 0.11±0.02 | 101±15 | 60.7 | ＞100 | | 300±50 |
